# Consequences of using chronological age versus corrected age when testing cognitive and motor development in infancy and intelligence quotient at school age for children born preterm

**DOI:** 10.1371/journal.pone.0256824

**Published:** 2021-09-02

**Authors:** Jacqueline F. Gould, Belinda G. Fuss, Rachel M. Roberts, Carmel T. Collins, Maria Makrides

**Affiliations:** 1 Women and Kids, South Australian Health and Medical Research Institute, Adelaide, South Australia, Australia; 2 School of Psychology, The University of Adelaide, Adelaide, South Australia, Australia; 3 Discipline of Paediatrics, The University of Adelaide, Adelaide, South Australia, Australia; Western University, CANADA

## Abstract

**Background:**

Children born preterm (<37 weeks’ gestation) have an increased risk of poor neurodevelopment, including lower intelligence quotient (IQ) scores compared with their term-born counterparts.

**Objective:**

To explore the differences in psychometric scores for cognition and motor skills when they are age-standardized according to chronological age instead of corrected age for children born preterm.

**Methods:**

We assessed = 554 children born <33 weeks’ gestation with the Bayley Scales of Infant Development, 2^nd^ edition (mental and motor scores) at 18 months and the Weschler Abbreviated Scale of Intelligence (IQ score) at seven years of age. Scores were standardized according to chronological age and corrected age and differences between mean chronological and corrected scores were compared, along with the proportion of children whose scores could be classified as impaired.

**Results:**

When scores were standardized according to chronological age instead of corrected age there was a large significant difference of 17.3 points on the mental scale (79.5 vs. 96.8, respectively) and 11.8 points on the motor scale (84.8 vs. 96.6, respectively) at 18 months. By seven years, the difference in IQ scores remained, although of a smaller magnitude at 1.9 points between mean chronological and corrected age scoring (97.2 vs. 99.1, respectively).

**Conclusion:**

Consistent with previous literature, outcome assessments for preterm infants consistently differed according to use of chronological or corrected age to standardized scores. Cognitive scores were impacted more severely than motor scores, and differences were more substantial in early childhood than later in childhood. For clinical purposes, correction for preterm birth is only likely to have an impact during early childhood, however assessments for research purposes should continue to correct into childhood to account for the persistent bias due to preterm birth.

## Introduction

Children born preterm (gestational age at birth <37 weeks) are at increased risk of experiencing impairment compared to their term born peers [1–5]. Children born preterm score significantly lower than their full-term peers on tests of cognition [3–6] and motor skills [3, 6], and are more likely to have neurodevelopmental deficits [1, 2]. The likelihood of a child experiencing a poor outcomes increases as gestational age at birth decreases [5, 7, 8].

Targeted clinical intervention may be the most promising method to minimise suboptimal child development in preterm samples [9–12]. Hence, it is routine practice in many developed countries to follow-up and assess the development of children born at the earliest gestations or with a low birth weight in order to facilitate early detection of deficits and assign intervention [13]. Developmental test scores are age standardized according to chronological age (calculated from date of birth) at time of assessment. However, there is debate as to whether corrected age (CA: adjusted for preterm birth, calculated from the estimated date of conception) is more appropriate for children born preterm, and if so, at what age is correction no longer necessary.

Age-standardized developmental tests are designed to indicate whether a child of a given age has the abilities typically seen in other children at that same age. Standardized scores contribute to a diagnosis of disability and are used to determine eligibility for services. In patients born preterm, use of chronological age to standardize scores may underestimate true abilities by comparing performance to older, more developed children. The likelihood of a score indicative of impairment hence increases and may contribute to unnecessary referral and/or diagnoses and excessive anxiety for a parent over a normally developing child. Alternatively, corrected age may overestimate performance and mask delay. This may post-pone appropriate diagnosis of impairment and prevent access to needed services.

Extensive literature has established that cognitive scores, such as intelligence quotient (IQ) scores, based on chronological age (uncorrected scores) are consistently lower than scores based on corrected age (corrected scores) [14–20]. Age-standardized scores for motor development appear to be similarly effected by preterm birth [21–24]. Modelling of scores on several cognitive and intelligence tests has highlighted that bias from using chronological age continues up to 16 years (the upper limit of the study), although the magnitude decreased with age and with lower scores [18]. When standardising scores of preterm-born children, use of corrected instead of chronological age may cause scores to differ by at least one standard deviation [15], particularly for the sub-group of preterm infants most vulnerable to disability, those born <29 weeks’ gestation [25, 26].

Correction for preterm birth appears to be particularly important for the test scores of young children [14, 18], while the magnitude of differences between corrected and uncorrected cognitive scores appear to diminish beyond three years age [18]. It has been hypothesized by some that children born preterm may eventually catch-up to their term-born counterparts, with motor abilities resolving at a younger age than cognitive abilities [25, 26], possibly as early as eighteen months of age [20, 23, 25]. However, there has not yet been an exploration of correction for preterm birth when standardizing both cognitive and motor abilities in the same sample [25, 26].

The aim of this study is to compare scores for cognitive and motor development that are age-standardized in the same sample according to both chronological and corrected age. We will explore the magnitude of the impact of correcting for preterm birth in early childhood and at school age in the same cohort, and whether there are implications for categorization of scores as average or indicative of possible impairment.

## Method

### Participants

This cohort of children was enrolled in a randomized controlled trial of docosahexaenoic acid supplementation, details published previously [27, 28]. A total of n = 657 neonates were enrolled at five major hospitals across Australia between 2001 and 2005; the Women’s and Children’s Hospital and the Flinders Medical Centre (Adelaide), the King Edward Memorial Hospital (Perth), the Royal Brisbane and Women’s Hospital (Brisbane), and the Royal Women’s Hospital (Melbourne). Infants were eligible if they were born less than 33 weeks’ completed gestation and were excluded from the trial if they had a major congenital or chromosomal abnormality, were from a multiple birth in which not all live-born infants were eligible, were in other fatty acid trials, or if fish oil was contraindicated [27, 28]. Data were collected at enrolment and at the assessments at 18 months and seven years’ corrected age.

The DINO trial and follow-ups were approved by each collaborating centre: Flinders Medical Centre—Southern Adelaide Clinical Human Research Ethics Committee (Application 221.11); King Edward Memorial Hospital–Women and Newborn Health Service Ethics Committee (Registration Number 1832/EW); The Royal Brisbane and Women’s Hospital—Queensland Children’s Health Services (RCH) Human Research Ethics Committee (Reference number HREC/ll/QRCH/7) and The University of Queensland Medical Research Ethics Committee (Project number 2012000021); The Royal Women’s Hospital–Royal Women’s Hospital Human Research Ethics Committee (Project 11/05); The Women’s and Children’s Hospital–Children Youth and Women’s Health Service Research Ethics Committee (REC2015/11/10). The parent or guardian of each participant provided written informed consent. The trial is registered with the Australian New Zealand Clinical Trials Registry: www.anzctr.org.au (ACTRN12606000327583).

As effects of the intervention on motor and cognitive development were negligible [27, 28], randomization groups were combined for the following analyses. Children were excluded from the present study if they performed below the threshold of the assessments, such as due to severe delay preventing valid administration of a test.

### Measures

Baseline data were collected at enrolment (shortly after birth) and children underwent assessments at 18 months and seven years [27, 28]. Participant characteristics such as maternal education and ethnicity were obtained from parents by structured interview at enrolment. Medical records were used to ascertain multiple pregnancy (twins, triplets), infant sex, gestational age at birth and birth weight.

The Bayley Scales of Infant Development, Second Edition (BSID-II) was used to assess participants at age 18 months and provided Mental Development Index (MDI) and a Psychomotor Development Index (PDI) [29]. The MDI assessed overall cognitive abilities including language, early problem solving, memory and early number concepts. The PDI assesses control of gross muscle groups (such as those required for standing, walking, running and jumping) and fine motor manipulations (such as those necessary for prehension and using writing tools) [29].

At seven years children underwent further cognitive testing with the Weschler Abbreviated Scale of Intelligence (providing a Full-Scale Intelligence Quotient [FSIQ]) [30]. The WASI is a brief (approximately 30 minute) assessment of intellectual functioning from four subtests (Vocabulary, Similarities, Block Design and Matrix Reasoning). There was no motor assessment at seven years.

Both the BSID-II and the WASI have scores that are age-standardized to have a mean of 100 and SD of 15. Age brackets for age-standardized scoring for the BSID-II are monthly at 18 months of age [29], whereas the WASI windows at seven years change every three months [30]. Children were classified as indicating likely impairment if the MDI or PDI score was less than 70 or if the FSIQ score was less than 90 (indicative of at risk of having a learning difficulty).

### Procedure

For the purpose of the original trial, appointments were booked according to corrected age, and age-standardized scores were corrected for prematurity. For the purposes of the present study, raw test scores were re-standardized according to child chronological age at the time of the assessment.

### Statistical methods

All analyses were performed in IBM SPSS Statistics, Version 24, and *P* values < .05 (2-tailed) were considered statistically significant.

Paired sample *t* tests were conducted to determine the degree to which corrected scores differed from uncorrected scores. Differences between corrected and uncorrected scores were recorded as new variables. McNemar Chi-Square tests were performed to investigate whether the correction of scores would lead to a different number of children identified as at risk for impairment compared with the use of uncorrected scores. At 18 months, scores were considered indicative of impairment if <70 (MDI or PDI scores) [29] as is typical in clinical practice. At seven years of age, we explored scores <90 (WASI FSIQ score) as scores of 90 or above are considered average or above [30] and are not considered a prerequisite for further follow-up, support or intervention. The movement between categories due to the use of correction was recorded and the Fisher exact test compared the movement of scores to determine whether the number of children moving categories and those remaining in their original category differed by domain. Additionally, a paired sample *t* test was performed to determine whether the mean difference in PDI score was different to the mean difference in MDI score.

## Results

**Fig 1:**
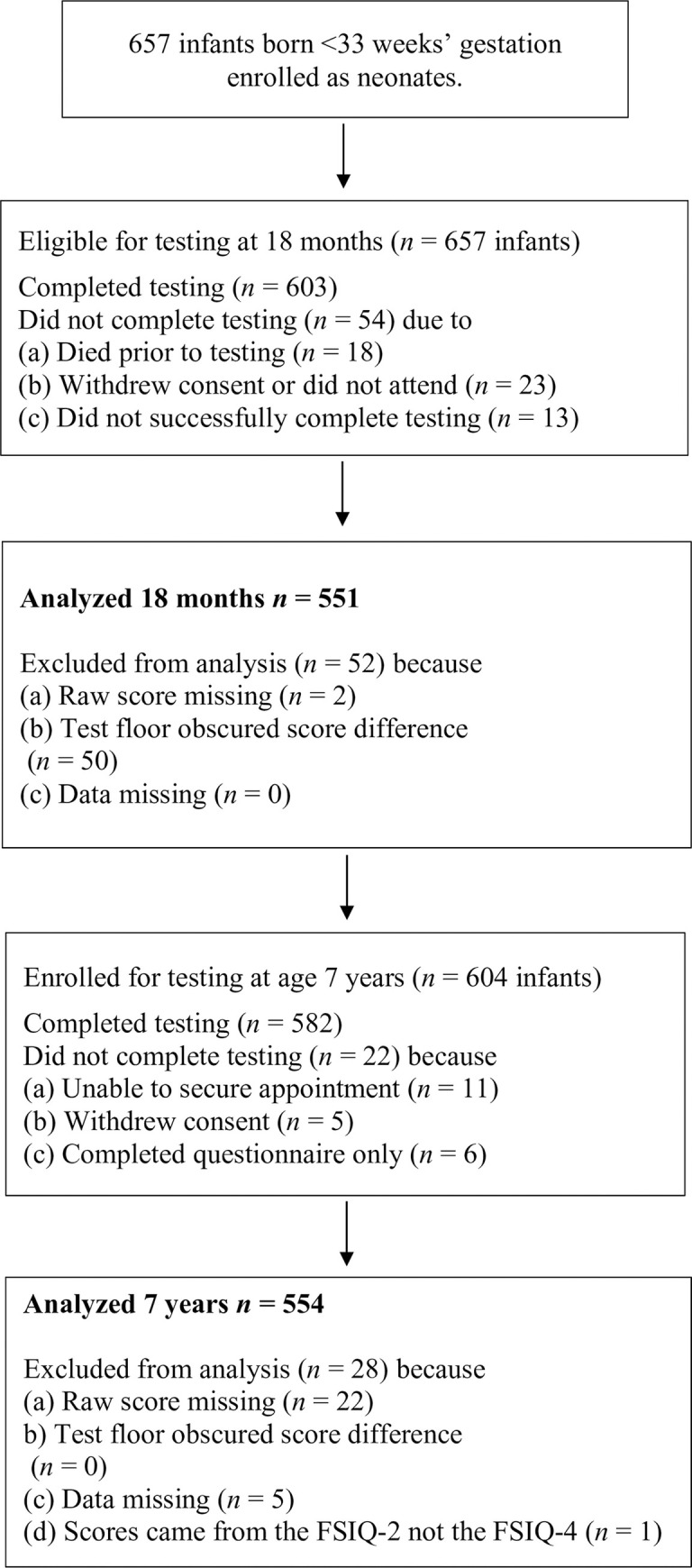
Flow diagram of participant assessments.

Of the 657 children enrolled in the original trial, 603 underwent testing at age 18 months, and 551 (91.38%) had useable BSID II data for the present study. There were 582 children who completed the WASI at seven years, and 554 (95.02%) had usable data for this study (Fig 1). No statistically significant differences in sex or ethnicity were evident between those excluded or included in the current analyses (see Table 1).

**Table 1:** Characteristics of those included and not included when children were eighteen months and age seven years of age.

Time-point	Prop. included	Prop. Excluded	*P* value
Ethnicity: Caucasian v. Non-Caucasian			
*18 months*	501/551 (90.1%)	49/52 (94.2%)	.424
*7 years*	508/554 (91.7%)	22/23 (95.0%)	.450
Sex: Female v. Male			
*18 months*	262/551 (47.5%)	22/52 (42.3%)	.472
*7 years*	260/554 (46.9%)	11/23 (47.8%)	.936

The sample was balanced in gender, with a substantial proportion (33%) born as part of a multiple birth, as is expected in preterm populations. The sample was overwhelmingly Caucasian (90%), with small proportions of Indigenous, Asian and other ethnic backgrounds (see Table 2).

**Table 2:** Key sample characteristics for children at age eighteen months and age seven years.

Characteristic	Age 18 months			Age 7 years		
	*n* = 551			*n* = 554		
GA mean, SD, range (wk)	29.3	2.3	23–33	29.3	2.4	23–33
Males, *n*, %	289	52.5%		294	53.1%	
Multiple pregnancy, *n*, %	187	33.9%		189	34.1%	
Had repeated a grade, *n*, %	25	4.5%		30	5.4%	
Ethnicity, *n*, %						
*Caucasian*	501	90.9%		508	91.7%	
*Aboriginal*	15	2.7%		16	2.9%	
*Asian*	23	4.2%		21	3.8%	
*Other*	12	2.2%		9	1.6%	
Maternal education^a^, *n*, %						
*Primary*	2	0.4%		3	0.5%	
*Junior Secondary*	80	14.5%		77	13.9%	
*Senior Secondary*	194	35.1%		184	33.2%	
*Tertiary*	274	49.7%		289	52.2%	
Chronological age, mean, SD, range (mo)	20.5	1.6	16–36	87.9	4.1	79–106
Corrected age, mean, SD, range (mo)	18.2	1.6	14–33	86.0	4.1	76–105

^a^ For 1 individual (0.2% of sample at both time points), data on maternal education was not registered.

**Fig 2:**
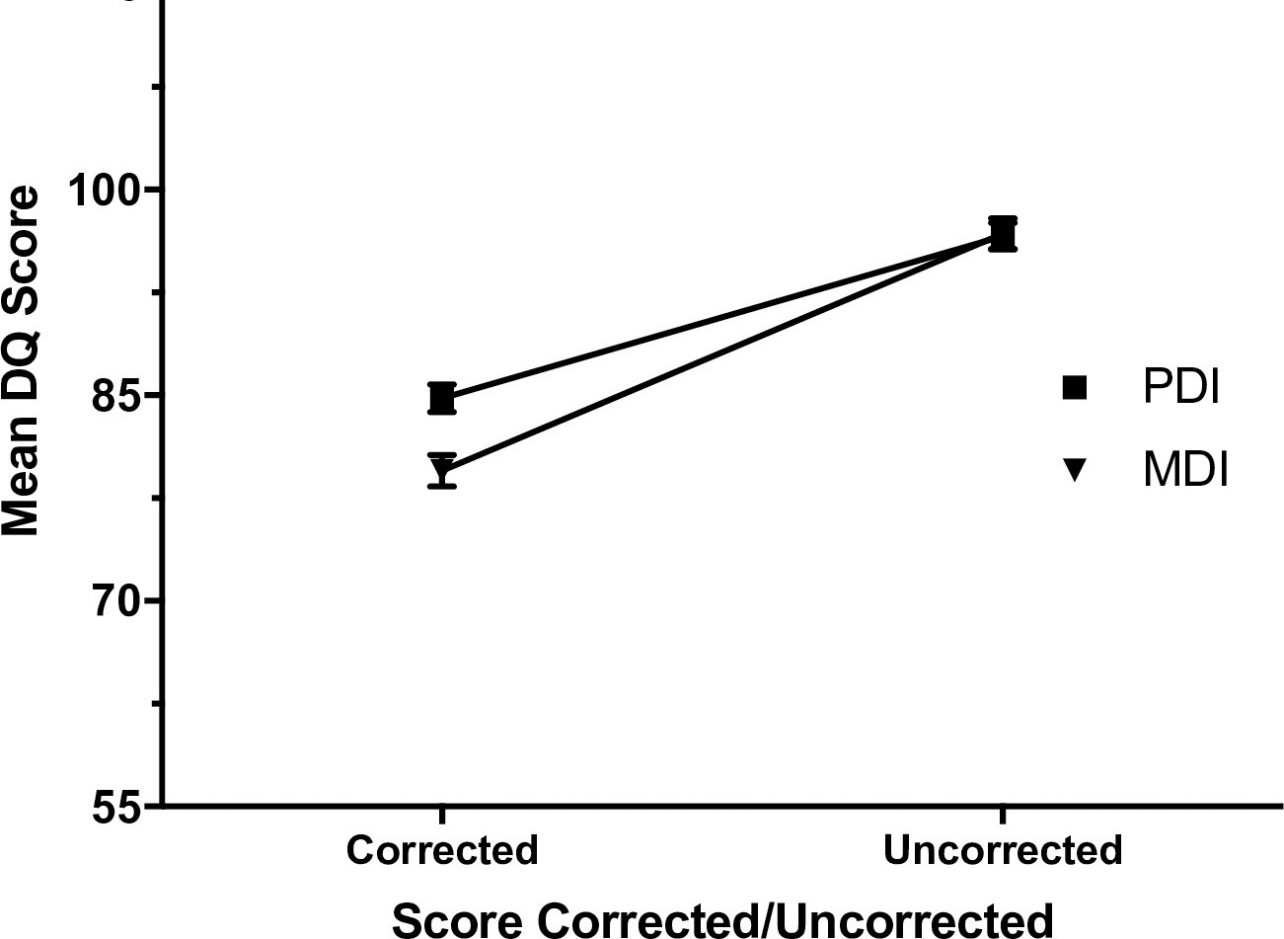
Visual depiction of Bayley Scale of Infant Development, second edition, mental development (MDI) and psychomotor development (PDI) index scores differ when corrected and uncorrected for preterm birth.

As anticipated, when children were tested at age 18 months their uncorrected PDI and MDI scores were lower than the scores following correction (all *P* values < .001; Table 3). The mean difference in MDI score was 17.3 points (range 2–33), whilst PDI scores differed by 11.8 points (range 1–30). The difference in corrected and uncorrected scores was significantly smaller for PDI (*d* = 0.93) than for MDI (*d* = 1.27; *P* < .001; Table 3, illustrated in Fig 2). Mean difference between MDI and PDI corrected and uncorrected scores was 5.5 points (range -7-21), where MDI scores were more significantly affected by correction for prematurity compared with PDI scores.

**Table 3:** Differences between scores on developmental tests that are age-standardized according to chronological age, and according to age corrected for preterm birth among children who were born <33 weeks’ gestation.

	Corrected	Uncorrected	Difference	Range	*P* value
18mo Bayley Scales of Infant Development *n* = 551					
MDI, *m* (*sd*)	96.8 (13.4)	79.5 (13.8)	17.3 (5.5)	2–33	< .001
<70, *n* (%)	16 (2.9%)	119 (21.6%)	103 (18.7%)		< .001
PDI, *m* (*sd*)	96.6 (11.6)	84.8 (12.0)	11.8 (4.4)	1–30	< .001
<70, *n* (%)	10 (1.8%)	52 (9.4%)	41 (7.6%)		< .001
7yr Weschler Abbreviated Scale of Intelligence *n* = 554					
FSIQ, *m* (*sd*)	99.1 (12.8)	97.2 (12.7)	1.9 (1.7)	0–8	< .001
<90, *n* (%)	134 (24.2%)	172 (31.0%)	38 (6.9%)		< .001

MDI Cohen’s *d* = 1.27.

PDI Cohen’s *d* = 0.93.

Difference Cohen’s *d* = 1.10.

MDI = Mental Development Index.

PDI = Psychomotor Development Index.

FISQ = Full Scale Intelligence Quotient.

FSIQ scores were slightly lower when uncorrected compared with scores that were corrected for preterm birth (mean difference 1.9 points, range 0–8; *P* < .001).

Most children were considered to have no likely motor (489 individuals, 88.7%) or mental (416 individuals, 75.5%) impairment at 18 months of age, and were unlikely to be at risk of having a learning difficulty at seven years of age (382, 69.0%) regardless of whether scores were corrected and uncorrected. When MDI scores were not corrected, 119 children (21.6%) were likely to have mental impairment and 86.6% of these children were no longer classified as likely to have an impairment after correction (*P* < .001). Similarly, uncorrected PDI scores resulted in 52 children (9.4%) being classified as likely to have a motor impairment, with correcting for preterm birth changing 80.8% to the no likely impairment category (*P* < .001). There was a larger proportion of children who changed category with correction for prematurity for the MDI, compared with the PDI (*P* < .001), however the majority of children did not change category regardless of correction for motor (499, 90.6%) or mental abilities (432, 78.4%). At seven years of age, 172 children (31.0%) were classified as likely to have a learning difficulty when scores were uncorrected, and 22.1% were no longer at risk after correction (*P* < .001).

## Discussion

Similar to previous cognitive [14–20] and motor [21–24] literature for correction of age-standardized scores, our sample demonstrated significant differences between all corrected and uncorrected scores and our effect sizes for all comparisons were large [31]. On average, cognitive scores at 18 months differed by more than a standard deviation but could differ by up to two standard deviations. At seven years the difference was much smaller (less than two points) but consistently resulted in a lower uncorrected score. Motor scores at 18 months of age were, on average, 0.7 of a SD higher when corrected for preterm birth but could also differ by up to two standard deviations. The magnitude of the differences seen at seven years are unlikely to change the clinical interpretation of underlying abilities, however at 18 months correction resulted in over 100 children being reclassified as being within the normal range for cognition, and therefore ineligible for further follow-up or intervention. The effect of correction on the clinical categorization of motor scores was likewise significant, with 40 children no longer falling below the cut-off for a possible impairment.

It is clear from our work, and from previous studies, that correction for preterm birth is likely to have a significant impact on the clinical interpretation of cognitive and motor test results in infancy, at least up to 18 months of age. However, assessments of cognition for clinical purposes likely do not need to be corrected beyond three years of age [18]. Future work will need to establish whether differences in motor scores from correction continue into childhood, although our sample suggests any differences are likely to be smaller than differences seen in cognitive scores.

Given that children born preterm are known to have lower scores for cognition and motor when compared with term-born children, even in the absence of impairment [5–7], it is unlikely that preterm children “catch up.” The smaller difference between corrected and uncorrected scores at older ages instead likely reflects that 1. neurodevelopment occurs rapidly between conception and three years of age with new milestones constantly emerging, and 2. That the windows for age-standardized scores are wider for older children. In the case of our study, the age bracket changed from one month at 18 months [29], to three months at seven years [30], making it less likely that a different score would need to be assigned at seven years for children born two months preterm.

The magnitude of the differences we detected are comparable to previous studies [14–24]. Although our study is the first with assessments at different ages in the same sample, our finding that the difference between corrected and uncorrected scores persisted at seven years of age (although of a much smaller magnitude than the difference at 18 months) is supported by previous findings of small differences beyond three years of age [14, 17, 18].

In light of the small, but persistent difference found in our sample up to the age of seven years, and through the statistical modelling of corrected and uncorrected cognitive scores up to 16 years of age [18], we recommend assessments conducted for research purposes are standardized according to corrected age, particularly in a sample consisting of both full-term and preterm children.

### Strengths and limitations

Our study has the unique advantages of both repeat assessments in the same sample, and a substantial sample (>500 at both time points). Assessment in infancy and at school age in the same sample means that differences in the magnitude of the effect of correcting for preterm birth at different ages can be reliably attributed to age and developmental progress of the sample. While correction has been investigated in regard to both cognitive and motor test scores, it is rare for studies to include differences for both simultaneously [20, 22]. To our knowledge, ours is the first statistical comparison between corrected and uncorrected motor and cognitive standardized scores, and the first to assess the same children at multiple ages.

Children were from multiple centres in Australia and the inclusion criteria were broad so that results are likely generalisable to the wider preterm population. The infants enrolled in the study were comparable to the population of infants in the Australian and New Zealand Neonatal Network at the time of birth [32, 33]. Unlike the majority of previous studies, all children in the current cohort were born after significant changes to clinical practice in neonatal units, notably the introduction of surfactant, are hence offer a slightly more contemporary comparison. The clinical management of infants born preterm continues to advance and has demonstrated benefits such as increased survival rates of those born at the earliest gestations. Medical advances have likely progressed since our cohort was born however, the cognitive outcomes of infants born preterm appear to have remained sub-optimal [3–5, 34].

The outcome measures we used were prevalent clinical tools at the time of the assessments, and the Bayley Scales are one of the most commonly tools used in routine neonatal unit follow-up and neurodevelopmental assessment of children under three years of age to identify developmental delay and infants requiring clinical intervention [13, 35, 36]. However, the BSID-II has been superseded by two editions (now in its fourth edition) and the WASI now has a second edition. The FSIQ of the WASI has a strong correlation with the FSIQ of the WASI-II [37]. The MDI of the BSID-II was separated into individual cognition and language scales although the overall motor score is similar [29, 38]. The current version of the BSID, with a distinct cognitive scale, is likely to be more similar to an early IQ score than the MDI. The mental score of the BSID-II has a moderate correlation with the cognitive and language scales of the third revision of the test [39]. Furthermore, it should be noted that the BSID, despite being one of the most commonly used assessments of early child development, is considered to have somewhat questionable predictive capacity for determining later child abilities, especially in preterm populations [12, 35, 40, 41]. Importantly given the results of previous studies, with differing assessments of cognitive and motor abilities, all reporting a similar magnitude of differences between scores based on corrected and chronological age [14–24] it is unlikely that results of a study using the current versions of the BSID or WASI would be significantly different. It is also noteworthy that although all children in our sample were born two months early, the majority scored within the normal range of development. Correction for preterm birth is likely to have a more significant impact on those born at the earlier gestations.

## Conclusions

The outcomes of the present study have implications for clinicians with patients that include children born preterm, as well as researchers. Our results support recommendations that researchers control for degree of prematurity when standardizing cognitive and motor scores throughout childhood. Assessments conducted for clinical purposes should be corrected for preterm birth at least up until 18 months of age, and likely up to three years of age, although clinicians should apply discretion in order to ensure that children are not excluded from accessing necessary support.
